# Surgical intervention for diffuse dermal angiomatosis of the breast

**DOI:** 10.1016/j.jdcr.2024.03.021

**Published:** 2024-04-15

**Authors:** Caroline E. Savoie, Bethany S. Acosta, Harley J. Davis, G. William Poche

**Affiliations:** a LSUHSC Department of Dermatology, New Orleans, Louisiana; b LSUHSC School of Medicine, New Orleans, Louisiana; c Martha E Stewart MD, LLC, Dermatology, Mandeville, Louisiana

**Keywords:** breast, cutaneous reactive angioendotheliomatosis, diffuse dermal angiomatosis, skin antiproliferation, vascular proliferation

## Introduction

Diffuse dermal angiomatosis (DDA) is a type of reactive skin angioproliferation. It is a benign, cutaneous vascular disorder that was first described in 1994 as a subtype of reactive angioendothelialiomatosis by Krell et al. While DDA is most often seen in the upper and lower extremities, several cases of DDA of the breast have been reported. DDA of the breast tends to affect individuals aged 40-60 years with multiple atherosclerotic risk factors such as hypertension, hyperlipidemia, diabetes mellitus, a chronic smoking history, and antiphospholipid syndrome.^1^, ^2^, ^3^, ^4^

Cases reported in the breast are commonly diagnosed in middle aged obese women suffering from breast hypertrophy. Although the pathogenesis of DDA of the breast remains unclear, many believe the weight of the pendulous breast, commonly occurring with obesity, leads to vascular compression and ultimately chronic tissue hypoxia. This can lead to upregulation of endothelial growth factors and the proliferation of new blood vessels to restore the diminished blood circulation and tissue oxygenation.^5^^,^^6^

DDA of the breast has a distinct clinical appearance. This rare disorder presents as red-violet purpuric papules and/or plaques with net-like violaceous vascular formations, complicated by nonhealing central ulcerations. Lesions tend to persist and progressively enlarge over time.^1^^,^^7^ Symptoms are usually severe intractable breast pain.^2^^,^^3^^,^^9^, ^5^, ^6^

Characteristic histologic features of DDA of the breast include a diffuse proliferation of endothelial cells and pericytes arranged between collagen bundles within the papillary and reticular dermis. Histopathologic examination also reveals subepidermal erythrocyte extravasation and diffuse dermal vessel proliferations with barely visible lumen. Immunohistochemically, the proliferating cells express endothelial markers such as ERG, CD31, and CD34.^7^, ^8^, ^10^^,^^10^

## Case report

In this report, we discuss a 58-year-old obese African American female with HIV and tobacco use disorder who presented for evaluation of a painful lesion on the right breast. Patient described a small red papule to the top of her right breast that initially appeared 4 months prior. She treated the lesion at home with topical combination of bacitracin, neomycin, and polymyxin B regularly, and it evolved into a nonhealing wound.

Examination revealed an obese woman with large pendulous breasts. Notably, there was a 1 cm ulcer to right breast with surrounding violaceous and hyperpigmented patches. Given possibility of external manipulation or contact dermatitis from antibiotic ointment, patient opted to forego biopsy and treat with basic wound care. She returned to clinic 2 months later and reported strict compliance with conservative care. No changes were noted on physical exam, and patient described interval progression in terms of pain and size. A punch biopsy was performed.

Initial biopsy showed a vascular proliferation in the dermis with adjacent dermal fibrosis. A moderate lymphocytic infiltrate with scattered dermal eosinophils were also observed. HHV-8 stain was negative, and FISH studies for MYC amplification were also negative. Given these results, the findings were deemed most consistent with diffuse dermal angiomatosis.

**Fig 1:**
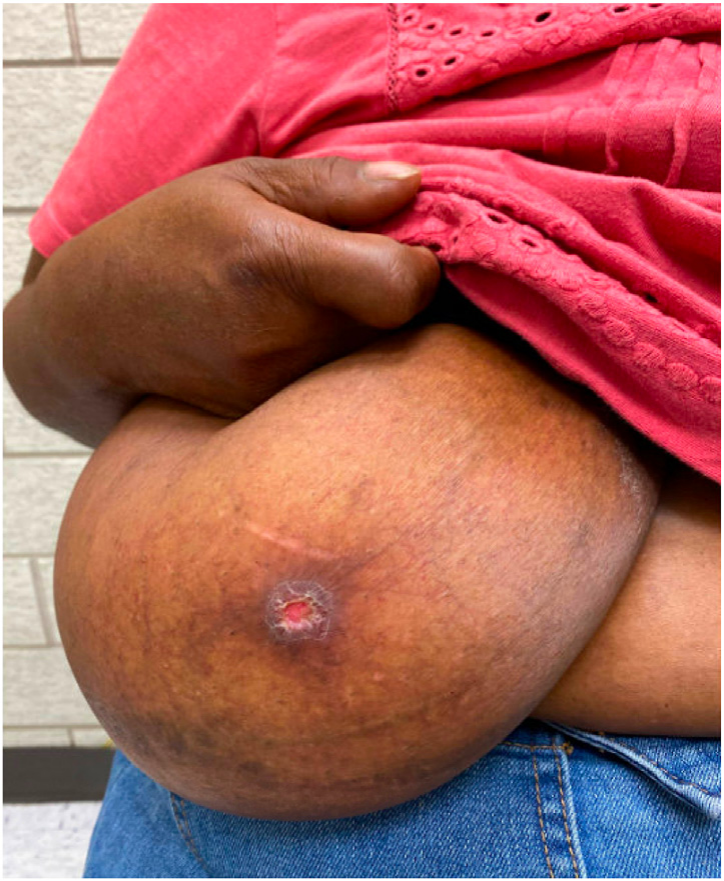
Right breast upon initial presentation with 1 cm ulcer with surrounding violaceous and hyperpigmented patches.

**Fig 2:**
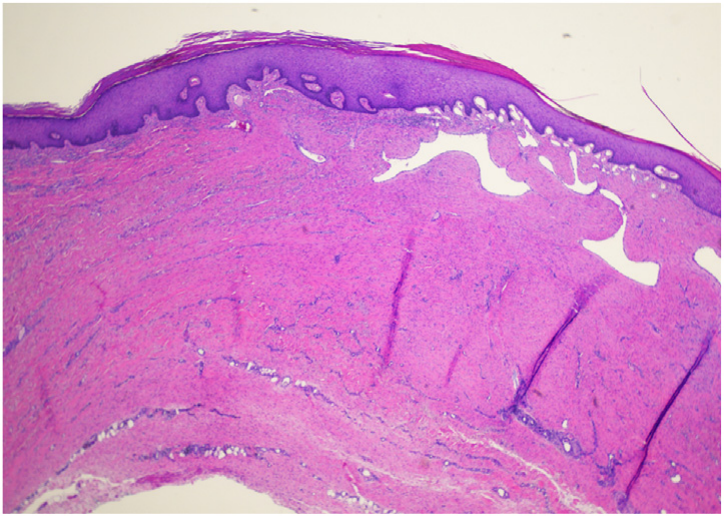
H&E at 2× magnification.

**Fig 3:**
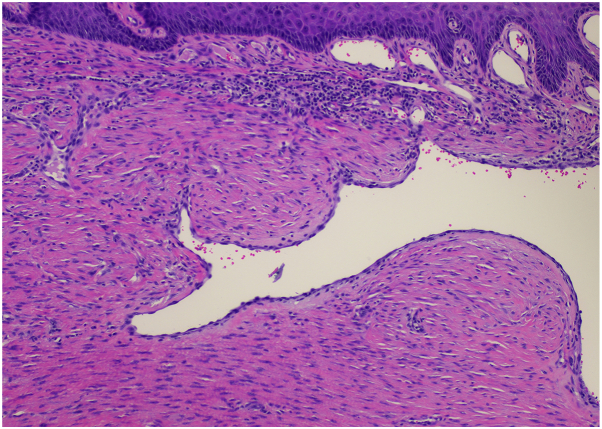
H&E at 10× magnification.

The biopsy site healed well, and additional management was initially deferred given patient preference for clinical monitoring. The patient returned 3 months later with complaints recurrence. Physical exam noted a 1.1 cm ulcer of right breast with granulation and fibrous tissue at the base of the lesion and surrounding lichenified skin (Fig 1, Fig 2, Fig 3). Excisional biopsy was performed. Again, the pathology report was consistent with diffuse dermal angiomatosis. Following full excision with a 3 mm margin around the ulcer site, the patient reported that the site fully healed, and she was no longer experiencing any associated pain. During assessment, more than 1 year after surgery, the patient remained with no recurrence of ulcer.

## Discussion

There remains paucity of data on the best treatment for DDA. Case reports have suggested a variety of interventions including isotretinoin, systemic corticosteroids, and even breast reduction surgery. Emphasis has been also placed on preventative measures to target tissue hypoxia such as smoking cessation and weight loss.^2^^,^^3^^,^^5^

Given concern for an underlying process producing recurrent ulceration in our patient, we proceeded with excisional biopsy. This removed the entire area of tissue with DDA.

While surgical resection showed promise in our patient, therapeutic intervention must be individualized for each patient and their circumstances. Given that DDA of the breast is frequently in the setting of large pendulous breasts and tobacco abuse, wound healing may be problematic. However, in the right subset of patients, excision of involved tissue could provide indefinite relief.

## Conflicts of interest

None disclosed.
